# Broadband, wide-angle antireflection in GaAs through surface nano-structuring for solar cell applications

**DOI:** 10.1038/s41598-020-63327-7

**Published:** 2020-04-14

**Authors:** Saraswati Behera, Paul W. Fry, Henry Francis, Chao-Yuan Jin, Mark Hopkinson

**Affiliations:** 1 Department of Electronic and Electrical Engineering, University of Sheffield, Mappin Street, Sheffield, S1 3JD UK; 2 College of Information Science and Electronic Engineering, Zhejiang University, Hangzhou, 310027 China

**Keywords:** Energy harvesting, Two-dimensional materials

## Abstract

We demonstrate broadband and wide-angle antireflective surface nanostructuring in GaAs semiconductors using variable dose electron-beam lithography (EBL). Various designed structures are written with EBL on a positive EB-resist coated GaAs and developed followed by shallow inductively coupled plasma etching. An optimized nanostructured surface shows a reduced surface reflectivity down to less than 2.5% in the visible range of 450–700 nm and an average reflectance of less than 4% over a broad near-infrared wavelength range from 900–1400 nm. The results are obtained over a wide incidence angle of 33.3°. This study shows the potential for anti-reflective structures using a simpler reverse EBL process which can provide optical absorption or extraction efficiency enhancement in semiconductors relevant to improved performance in solar photovoltaics or light-emitting diodes.

## Introduction

Using surface nanostructures to obtain antireflection properties for improved absorption or light extraction are highly applicable to thin-film solar cells and light-emitting diodes^1–5^. GaAs are widely used in semiconductor optoelectronics for lasers^6^, LEDs^7,8^ and solar cells^9,10^. It is almost a preferred material for solar cell applications due to its high conversion efficiency under low light conditions and high thermal conductivity, making it useful for spacecraft or aircraft. However, one of the key issues for these applications is the large refractive index of the semiconductor which leads to a surface reflectivity of over 35% in the visible range. To address this problem, antireflective coating layers are employed which have achieved a solar to electrical conversion efficiency up to 28% for GaAs-based single-junction thin-film solar cells^11^. However, although thin antireflection layers or multilayer coatings reduce surface reflection up to a certain wavelength range, they show peak antireflection only for a narrow wavelength band of light based on the thickness of the material. Antireflection coatings show directional dependent properties according to the angles of incidence and polarization. This may lead to a loss of energy in the coating region either due to total internal reflection (TIR) over critical angles or due to material absorption.

Active/absorbing material surface nanostructuring is an alternate scheme to achieve electromagnetic energy trapping for broadband device applications in terms of optical absorption over a wide incident angle, which will contribute to the external quantum efficiency of solar cells and the extraction efficiency in LEDs^12,13^. Single junction materials, such as Si or GaAs have reached a high degree of refinement. Further enhancement of the solar conversion efficiency is unlikely to come from materials factors and instead antireflective surface nanostructuring for absorption enhancement and light trapping offers a more worthwhile route. There have been several interesting studies on this aspect^14–18^. Surface nanostructuring studies reported in^14^ by Kim. *et al*. show an average surface reflectance of more than 10% for 400–800 nm wavelength range, whereas the studies present by Han *et al*.^15^ report an average reflectance of more than 5% over the 300–800 nm wavelength range. Lower reflectance of ~3% is reported by Liang *et al*.^16^ for the 440–880 nm wavelength range. However, the thickness of the features within the antireflection layer is relatively large with a 900 nm height and a 650 nm diameter. These relatively deep features require more material and fabrication time, which will translate into higher costs. Although all these results are promising, there remains a significant opportunity to achieve surface reflectance reductions down to a few percents over a broadband optical wavelength range and over wide angles for efficiency enhancement in GaAs based solar cells following comparatively simpler fabrication steps.

Surface nanostructuring in semiconductors has been undertaken using nanosphere lithography^14^, nanoimprint lithography^15^, electron beam lithography (EBL)^19^, and interference lithography^20^ which have produced binary and motheye types of surface nanostructuring. In each surface patterning process, reproducibility, resolution, patterning cost, patterning time and design flexibility are the key parameters for robust applications. Among all the techniques, laser interference lithography is a time-effective, large area and cost-effective technique for micro/ nano-scale patterning for semiconductor materials and subwavelength scale patterning for antireflection coatings^20–26^. However, in this case, minimum features are limited by the diffraction limit of the laser. Uniformity, periodicity and precise control over feature sizes are important in semiconductor nanofabrication for broadband, wide-angle and polarization-independent applications. Achieving exact exposure for the realization of features as per the design demands many trials or optimization of photoresist and exposures.

EBL^27–29^ is the most precise of the techniques and has the capability to achieve simple or complex high-resolution surface nanostructuring of semiconductors. However, even using this method it is possible that the pattern achieved differs from the intended structure due to variables such as the electron beam (e-beam) dose and inductively coupled plasma (ICP) etching rates. These variations can lead to a significant variation in the optoelectronic properties. For constant EBL exposure and developing time, a variation in ICP parameters changes the features and vice-versa. Therefore, it’s important to understand the effect of e-beam dose and ICP etching rate by keeping one parameter constant and by varying the other in a manner to obtain optimized structures for enhanced optoelectronic properties.

In this paper, we demonstrate surface reflectivity minimization in GaAs through a reverse EBL based nanostructuring process and compare the experimental observations with simulations. Note, our use of EBL may not be applicable to the final application due to cost and throughput consideration. Rapid, cost-effective processes such as interference lithography may be more suitable for real-world applications. However, EBL provides us with an accurate fabrication process with which we can achieve the type of sub-wavelength structures we have designed and simulated by FDTD. Using this approach, we have been able to reduce surface reflectance to less than 2.5% over a broad wavelength range of 450–700 nm. The subwavelength features with parabolic sub-wavelength scale-space variations with subwavelength axial optical path provide a phase change over the interface that leads to the condition of minimum intensity, in a similar manner to motheye kind of surface variations leading to antireflection properties.

We apply variable patterning parameters to the GaAs structure using EBL followed by ICP etching. This technique has an advantage over other techniques as it offers the possibility of exposing multiple doses over a single pattern in a controlled manner to obtain high contrast surface patterning for a constant etch rate. We have studied the effect of using simple steps and various depth profiles of the textured GaAs interface along with surface modifications based on the dose parameters in a single exposure. We report a scanning electron microscope (SEM) surface analysis of the fabricated samples and optical reflectance studies. After ICP etching, we find dramatically reduced surface reflection in the visible wavelength range from 450–700 nm. We have achieved a minimum surface reflectance of less than 2.5% in the visible wavelength range for an optimized e-beam dose and using an ICP etch time of 2.15 minutes. We propose that this kind of surface texture can be applied either to light-emitting diodes or to solar photovoltaics to enhance light extraction or light trapping, respectively. Here we concentrate on the effect of surface reflectivity. We have carried out FDTD simulation studies on the optical properties such as reflectance and total absorbed power for designed reverse patterned GaAs solar cells to validate the experiment and show possible applications towards solar energy harvesting.

## Experiments and data analysis

### Surface analysis

**Figure 1:**
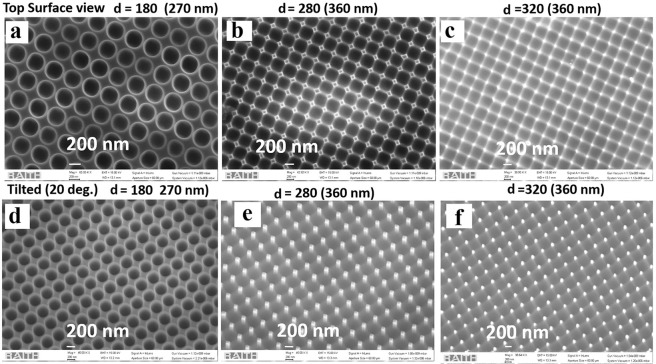
SEM images of the fabricated and etched GaAs sample top surface view (**a**) designed with a dimeter of d = 180 nm and realized diameter of the parabolic air holes after etching is 270 nm, (**b**) designed dimeter (**d**) of 280 nm leading to holes of 360 nm, designed diameter (**d**) of 320 nm leading to 360 nm and (**d**–**f**) shows 30° tilted view of the images in (**a**–**c**).

**Figure 2:**
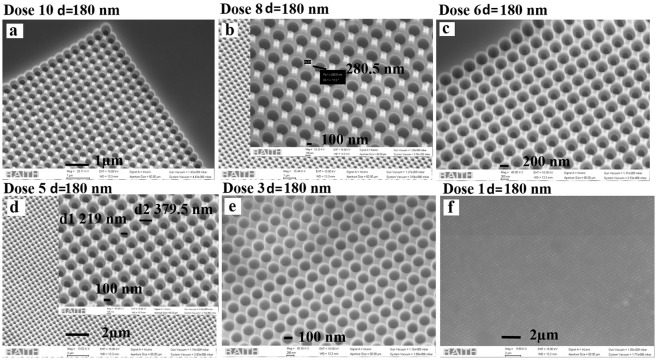
SEM images of the etched GaAs sample for a constant diameter (180 nm in design) and etch time (2.15 min) on different EBL doses (**a**) dose 10, (**b**) dose 8, (**c**) dose 6, (**d**) dose 5, (**e**) dose 3 and (**f**) dose 1.

**Figure 3:**
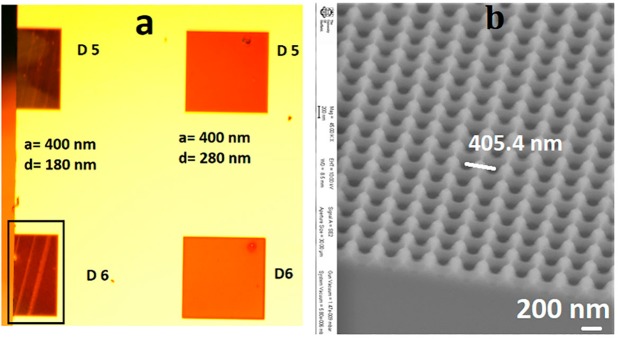
(**a**) Shows optical microscope image of the sample containing several doses and design parameters, (**b**) shows cross-sectional SEM image of the sample having dose 6 with design parameters, periodicity, a = 400 nm, and diameter d = 180 nm presenting parabolic kind of depth variation over the cross-section The corresponding top surface view of the dose 6 sample is presented in Fig. 2(c).

**Figure 4:**
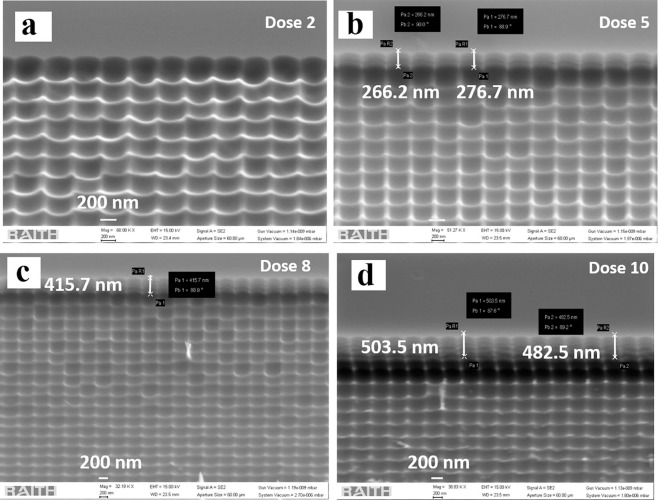
(**a**–**d**) SEM images showing the cross-sections of the sample with design parameters periodicity, a = 400 nm and d = 280 nm over different doses showing different axial depths varying from dose 2 to dose 10.

Here we present surface morphology analysis of the fabricated nanostructured GaAs substrate through EBL carried out at an acceleration voltage of 50 kV and a beam current of ~200 pA. A range of exposure doses was used starting at 70 µC/cm^2^ (dose 1) and increasing in increments of 7% up to a maximum of 1.8 times of the initial dose (dose 10). SEM analysis of the samples with different e-beam doses (1–10), after ICP etching for 2.15 minutes is presented in Figs. 1–4. In all the figures there exist parabolic holes of different diameters. In Fig. 1(a) the sample had a design parameter of d = 180 nm, but we obtain a diameter of 270 nm experimentally after ICP etching. Similarly, we obtain a diameter of 360 nm for a theoretical design of 280 nm in Fig. 1(b). With a further increase in the diameters, to 360 nm, the sidewalls collapse, and we are left with small pillars of few nm height as observed in Fig. 1(c). The corresponding 30° tilted views of the SEM images are presented in Fig. 1(d–f). Simulation has shown that these small pillars can give rise to undesirable surface reflection or diffraction effects which can interact with the contribution from subwavelength scale holes. The design with d = 180 nm which leads to well-separated gradient parabolic holes as shown in Fig. 1(a,d) is the most suitable for reduction in surface reflectance. Therefore, we have concentrated on these smaller dimensions and moved on to analyze the effect of different doses on the surface features.

The SEM analysis of samples with different dose factors for a specific design is shown in Fig. 2. We could obtain a well-separated parabolic hole array, but with some interconnected regions removed showing the presence of small nano features in-between as shown in Fig. 2(a,b). Decreasing the dose factor gradually reduces the exposure and, as a result, we see well-separated parabolic holes for doses 3–6 as evidenced from Fig. 2(c,d). For doses 5–6, these lead to a dual cone/diameter type of parabolic profile with an external diameter of approximately 379 nm and an internal diameter of 219 nm. This gradual variation in diameter and parabolic height profile is expected to reduce the surface reflectance to a lesser extent compared to a planar interface. A reduction in dose leads to uniform parabolic holes with uniform single diameter is presented in Fig. 2(e) and a further reduction in EBL charge density lead to very shallow patterns of only a few tens of nm as shown in Fig. 2(f). In these samples the surface reflection is bulk-like.

It is observed in Fig. 2 that doses 5 and 6 based-nanostructuring over GaAs have similar surface morphology and features. Therefore, we show a cross-sectional view of the SEM image for the sample with dose 6 using a Loomis scribe and break machine. This shows the parabolic depth profile of the samples that lead to gradient refractive index change over the interface as presented in Fig. 3 ^30,31^. The surface reflectance from the patterned structures is also dependent upon the etch depth profile^2^. Therefore, we have also studied the effect of different etch depths with different e-beam doses. We observe, based on the beam current of the electron beam doses, the etch rate has been affected leading to different etched depths for a constant span of etching time. The effect of EBL dose parameters on the depth profiles is presented in Fig. 4 through tilted view SEM images. With an ICP etching time of 2 minutes, doses 1–2 give a shallow depth of less than 100 nm, dose 5 gives a depth of approximately 276 nm, dose 8 gives 415 nm and dose 10 a depth of approximately 482 nm as shown in Fig. 4.

### Reflectance measurement

**Figure 5:**
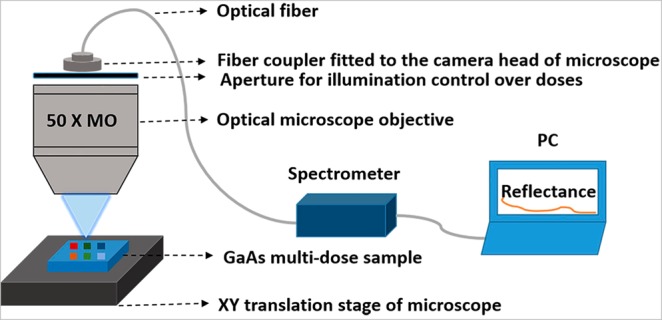
Experimental setup to measure wide-angle reflectance of the sample of different doses, diameter, and pitch over a single wafer in a controlled manner (we have shown only the objective part of the microscope for simplicity).

To characterize the multi-dose and multi-parameter microscale patterned regions of our sample, we use a Silicon CMOS array detector spectrometer that is integrated to an optical microscope and provided with visible illumination (450–700 nm) as shown in Fig. 5. In this measurement, the reflectance of each pattern which contains different doses, diameters, and periodicities is obtained. By ensuring the precise movement of the sample position through the translation stages we can accurately measure each pattern through the microscope. The illumination area is limited to specific patterned regions through aperture control of the microscope. Furthermore, this setup can collect the reflected signal over a wide-angle with the use of a high numerical aperture up to 1 or with a 100 X microscope objective (MO). We have used a 50X MO with NA = 0.56 in our case for our study that covers a half cone angle of 33.3°. We have compared the surface reflectivity of the patterned substrate concerning an un-patterned substrate for several doses of e-beam exposure under a constant ICP etching time.

**Figure 6:**
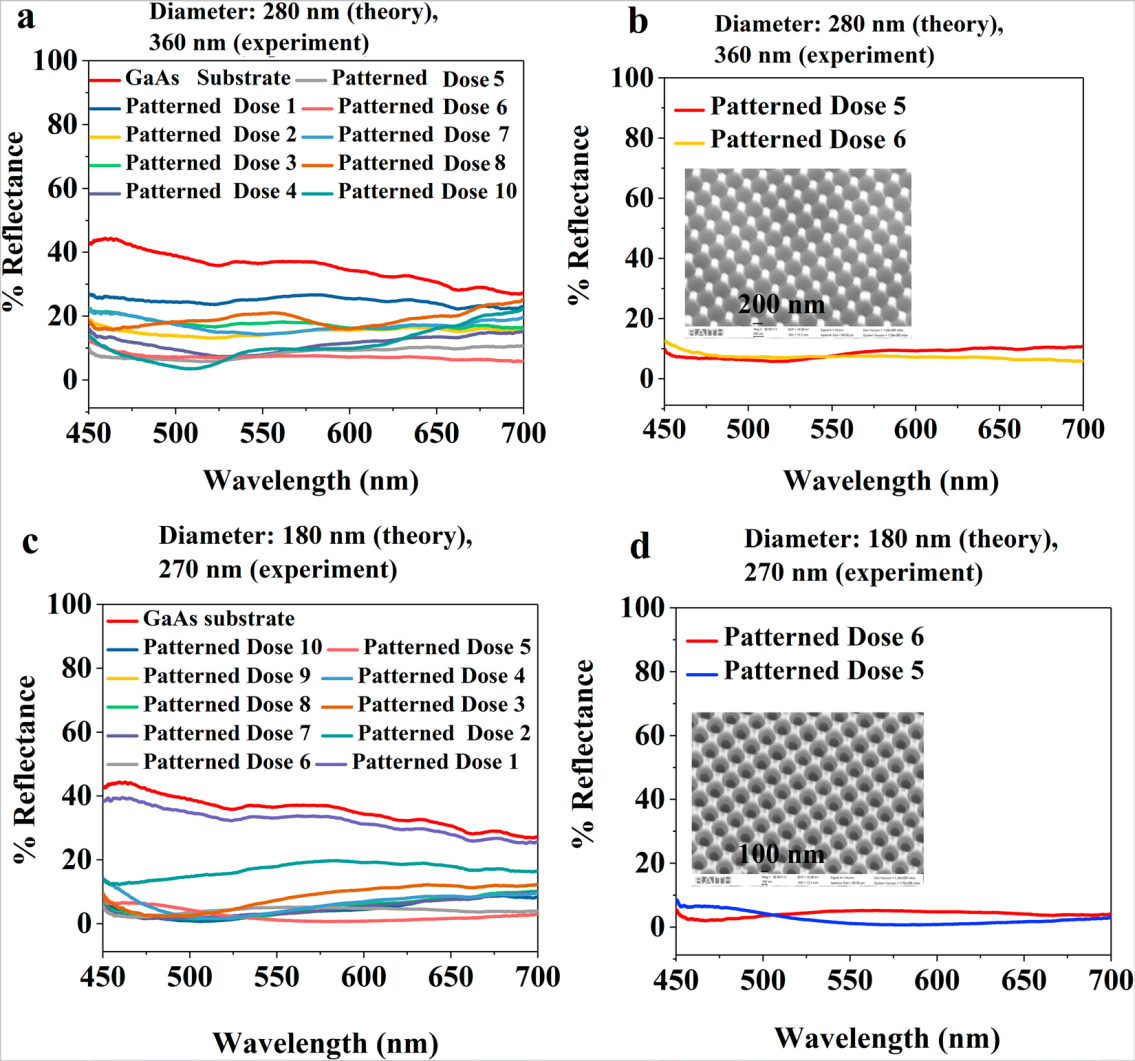
Measured surface reflectance of the textured GaAs sample (**a**) with variable electron beam doses (1–10) for diameter 280 nm (theory) or 360 nm (experiment), (**b**) diameter 280 nm (theory) or 360 nm (experiment) with doses 5 and 6 showing minimum reflectance of 7.35% over 450–700 nm wavelength range, the SEM image in inset corresponds to that of the dose 5 as shown in Fig. 4(b,c) diameter 180 nm (theory) and 270 nm (experiment) and (**d**) diameter 180 nm (theory) or 270 nm (experiment) with doses 5 and 6 showing minimum reflectance of 2.5% over 450–700 nm wavelength range, the SEM image in the inset shows that of the dose 5 presented in Fig. 2(d). Here, theory refers to the design by EBL Raith software and the experiment refers to the experimentally observed pattern through EBL.

The observed reflectance spectra are presented in Fig. 6. The measured reflectance from the bare GaAs substrate is as high as 39% percent in this wavelength range due to the high refractive index contrast between air and the dielectric medium (*n* = 3.8). The textured surfaces as shown in Figs. (1–3) show a significant reduction in refractive index contrast through a gradient variation due to the subwavelength patterning features that have reduced the surface reflectance. By varying the dose of the e-beam the size of the holes in the resist changes. Larger hole sizes lead to an increased etch rate for constant ICP parameters and a variation in the etch depth is observed during this experiment. For an obtained diameter of 360 nm through EBL, the depth variation due to the ICP rate affects the surface reflectance as shown in Fig. 6(a,b). A shallow depth (approx. 100 nm) for dose 1 after ICP leads to a small reduction in the surface reflectance. For dose 5, the ICP rate changes leading to approx. 280 nm depth profile as shown in Fig. 6(b), we see the reflectance gradually reduces and it becomes minimum for dose 6 (7.35%).

A further increase in depth using higher doses increases the surface reflectance. Therefore, it is concluded that for subwavelength features or a depth (path length) of 200–300 nm, there is a minimum in the surface reflectance which takes place due to the phase change at the air/patterned semiconductor interface. We have also carried out a study with different doses of an EBL leading to the different diameters and observed the effect of etch rate on depth profile affecting surface reflectance as shown in Fig. 5(c,d). It is observed that a theoretically designed diameter of 180 nm, which corresponds to an experimentally observed diameter of 270 nm has reduced the surface reflectance drastically for certain doses. As evidenced from Fig. 6(c), the reflectance reduces from 39% to 2. 5%. As shown in Fig. 6(d), Dose 6 shows a 6.25% surface reflectance, and this further reduces to 2.5% which is flat over a broad 450–700 nm wavelength range for dose 5 of electron beam exposure. Thus, it is concluded that reverse surface parabolic texturing can also reduce the surface reflectance in GaAs over a broad wavelength range and over wide incident angles enabling it suitable for high light extraction or light-trapping/ absorption applicable in light-emitting diodes or solar cells.

**Figure 7:**
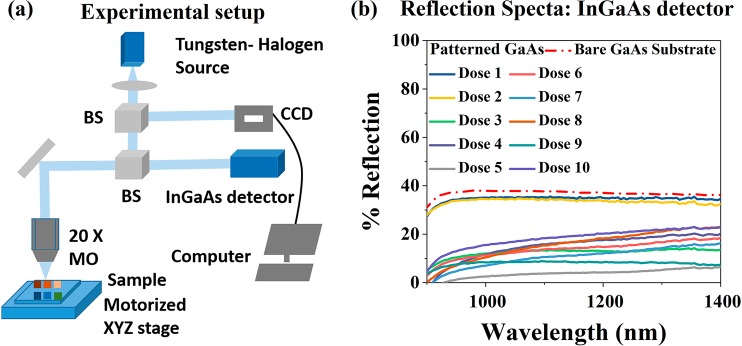
(**a**) Experimental setup to measure reflectance of the samples of different doses, diameter, and pitch over a single wafer through a NIR InGaAs detector using a micro-photoluminescence (µ-PL) based optical setup with motorized controlled stages and (**b**) measured reflectance over 900–1400 nm for several doses of the EBL patterned and etched samples. MO: Microscope objective, M: Mirror and BS: Beam splitter.

To study the reflectivity in the near-infrared range, we have carried out a separate experimental study using the experimental setup presented in Fig. 7(a) using an InGaAs detector. Using this set up we can measure the surface reflectance of the samples from 900–1400 nm wavelength range as presented in Fig. 7(b). It is observed that a bare GaAs substrate shows an average reflectance of 36% from 900–1400 nm, whereas the surface nanopatterning has drastically reduced the surface reflectance. We observe an average reflectance of 4% for dose 5 over a broad wavelength range from 900–1400 nm. Therefore, from the above study, we found that reverse surface patterning in GaAs has highly reduced the surface reflectance to less than 2.5% and increased the surface absorbance over a broadband wavelength of 450–700 nm visible wavelength range, which is important for efficiency enhancement in GaAs based solar cells.

### FDTD simulation

**Figure 8:**
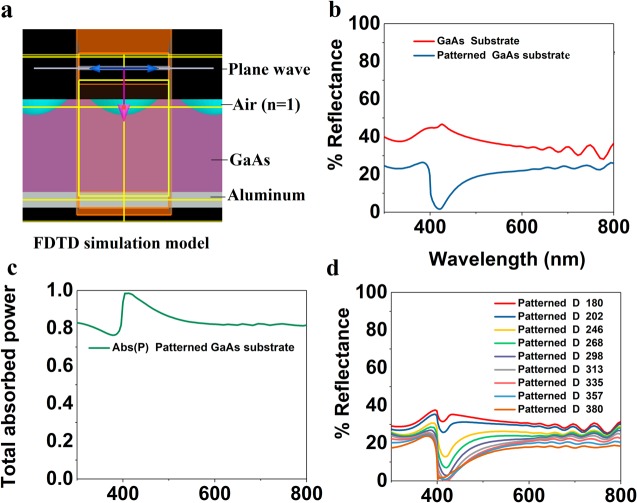
(**a**) FDTD simulation model in Lumerical, (**b**) simulation studies on the reflectance of the sample (**c**) comparison between bare GaAs substrate and patterned GaAs substrate with a parabolic air hole of diameter 300 nm, (**c**) Calculated total optical absorbed power due to the solar photovoltaic module and (**d**) minimization study and comparison of the reflectance studies between different patterned substrates with variable diameter of parabolic air holes from 180 nm–380 nm.

To verify experimental results and show possible applications, we have carried out 3D FDTD simulation studies to understand the minimization of reflectance from textured GaAs substrate using a reverse patterning process. As shown in Fig. 8(a), we have carried out FDTD simulation studies on the model of GaAs substrate (pn junction solar cell) of 1.2 µm thick with a reverse surface nanostructuring of periodic square lattice-based parabolic air holes of 400 nm periodicity, 300 nm diameter and 200 nm depth with a 200 nm thick Aluminum back reflector. We have considered a unit cell for simulation with periodic boundary conditions along the X-Y plane and perfectly matched layers along Z-axis and normal incident plane-polarized light. Such a study can also be made for different polarization angles and for a wide angle of incidence. We have considered GaAs material from the material database of Lumerical with optical constants for broadband wavelengths where, the real part refractive index (n) = 3.856 and extinction coefficient/ imaginary part of refractive index (k) = 0.196 at 632.8 nm; n = 4.12 and k = 0.32 at 532 nm. The bare substrate shows an average of 38% reflectance over 400–700 nm that is reduced to 16% through patterning of the GaAs surface as observed from Fig. 8(b). A deep is observed in the simulated reflection spectrum at a wavelength of around 420 nm. This arises due to the complete absence of photonic modes at this wavelength that are transmitted to the substrate based on the designed parameters. This absorption in GaAs due to our photonic design is dominant over the typical absorption band of GaAs material. At present we don’t have a UV detector that can experimentally detect the deep in UV region of the spectrum. We have used the solar generation analysis module of FDTD solutions to calculate the total power and solar generation efficiency through simulation. We observe a total absorbed power of 83% over 300–800 nm as shown in Fig. 8(c). Furthermore, we have carried out the comparison study between reflectivities due to different diameters of the air holes to optimize the suitable diameter for minimum reflectance as shown in Fig. 8(d). It is observed that the reflectivity gradually reduces for the increase in diameter of the holes from 180 nm until 380 nm and could be reduced up to 12.3% for a diameter of 380 nm is presented in Fig. 8(d). We observe all the reflectance for normal incident light in case of a simple parabolic reverse profile.

**Figure 9:**
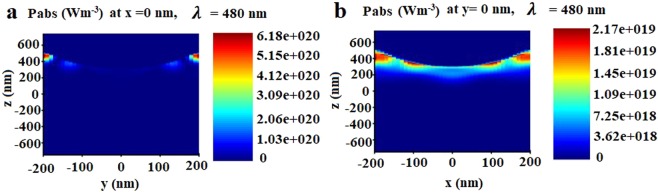
FDTD simulation results on the (**a**,**b**) absolute power along YZ and XZ plane due to reverse textured GaAs substrate at 480 nm (peak absorption wavelength).

Although simulated results show the reduction of surface reflectivity in GaAs due to effective reverse surface nanostructuring, the reduction in the reflectivity is comparatively lower than the experimentally observed results. The difference we believe is due to a complex profile derived from the ICP etching which results in further light trapping. The absolute absorbed power in the normal Z (XZ and YZ) planes inside the GaAs substrate due to the reverse patterning at GaAs interface is presented in Fig. 9(a,b). This simulation study indicates that surface patterning in GaAs has drastically reduced the surface reflections and increases the total optical absorption power (83%) in the GaAs substrate over a broad wavelength range (450–700 nm) and the maximum power absorbed through the volume of the substrate is 6.8 × 10^20^ Wm^−3^. Therefore, this texturing can find application in efficiency enhancement in solar photovoltaics towards broad-spectrum solar energy harvesting.

## Methods

### EBL- multi-dose surface nanostructuring

**Figure 10:**
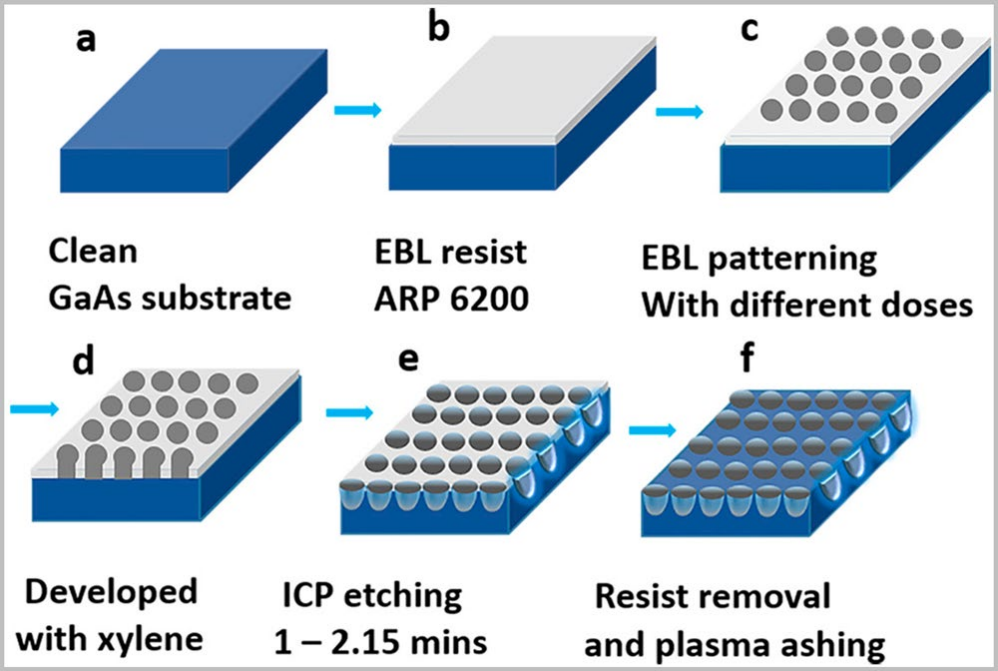
Schematic of the experimental steps, (**a**) substrate cleaning, (**b**) resist coating, (**c**) EBL patterning with variable doses, (**d**) developing, (**e**) ICP etching and (**f**) resist removal and plasma ashing.

Surface nano-patterning of GaAs has been undertaken using EBL followed by ICP etching as per the schematic presented in Fig. 10. Square lattice arrays with a range of design parameters and dose variations were designed using proprietary Raith GmbH pattern editing software. The design contains periodic square, hexagonal and honeycomb lattice arrays of varying diameters 180 nm, 240 nm and 320 nm with a periodicity of 400 nm for the square lattice. For the hexagonal lattice, periodicity along the X-axis is 400 nm and along Y-axis is 346.41 nm. For the honeycomb lattice, periodicity along X-axis is 800 nm and along the Y-axis is 692.82 nm with quasiperiodic or long-range spatial periodicity.

A clean GaAs substrate was spin-coated with a 200 nm thick film of the positive electron beam resist AR-P-6200 and post-baked at 180 °C for 3 minutes to remove the liquid residues. The pattern was exposed with a Raith Voyager electron beam writer at an acceleration voltage of 50 kV and a beam current of approximately 200 pA. Exposure doses ranged from 70 µC/cm^2^ to approximately 130 µC/cm^2^. Exposed samples were then developed in a xylene solvent for 1 min, followed by rinsing in iso-propyl alcohol (IPA). The developed pattern in the positive resist shows holes of variable diameters and features depending upon doses and design parameters. The reverse photoresist patterned GaAs substrate was etched using ICP etching for 2 or 2.15 minutes. The etching parameters were as follows: Chlorine (Cl_2_) – 20 sccm; Argon (Ar) – 10 sccm; RF power – 200 W; ICP power – 300 W; pressure – 2mTorr. A decrease in hole size results in a slower etching rate of the GaAs which gives rise to different features and depths for different doses in the sample. The transferred pattern presents with diameters varying from 220–380 nm and depths varying from 100 nm to 482 nm. The residual resist is then removed from the substrate using Microposit 1165 resist remover followed by cleaning with acetone and IPA. Finally, the sample is ashed with an oxygen plasma for 5 minutes.

### Summary of structural and optical properties

We present a summary on the structural and optical properties of the realized nanostructured GaAs substrate with several feature sizes using variable dose-based EBL patterning and ICP etching technique in Table 1.

**Table 1:** Summary of structural and optical properties.

Material/nanostructure with doses		Average reflection in % over a broadband wavelength range	
**1. Bare GaAs substrate (100)**			
35% 450–700 mm, 36% 900–1400 nm			
**2. Nanostructured GaAs with variable EBL exposure doses**			
EBL design parameters	Experimentally observed parameters		
(i) a = 400 nm, d = 180 nm (ii) a = 400 nm, d = 280 nm	Periodicity (approx. in nm) (i) 405 (ii) 405	Diameter (approx. in nm) (i) 270 (ii) 360	Depth (approx. in nm) (i) 220 (ii) 260
Ebeam Doses	% of Reflection for (i) and (ii)	Wavelength ranges in nm	
Dose 1	31 (ii) 24 34	450–700 900–1400	
Dose 2	16 (ii) 15 33	450–700 900–1400	
Dose 3	8 (ii) 16.23 12	450–700 900–1400	
Dose 4	6.2 (ii) 11.3 15	450–700 900–1400	
Dose 5	**2.5** (ii) **8.4** **4**	450–700 900–1400	
Dose 6	4.1 (ii) 5.1 13	450–700 900–1400	
Dose 7	5.1 (ii)17.9 10	450–700 900–1400	
Dose 8	5.1 (ii)19.05 15	450–700 900–1400	
Dose 9	5.4 (ii)12 8	450–700 900–1400	
Dose 10	4.5 (ii)10.9 18	450–700 900–1400	

## Conclusion

We have carried out experimental and simulation-based studies to demonstrate broadband and wide-angle antireflection on high reflectance GaAs substrates. Through inverse e-beam patterning, we have controlled the electron beam charge density parameter for a constant ICP etching time. This has led to different etch rate controlled variable features. We achieve broadband reflectance down to 2.5% for a 450–700 nm visible wavelength range and less than 4% for 900–1400 nm NIR wavelength range over a wide angle of 33.3°. We believe this optimized study of EBL conditions can help researchers in understanding the exact dose parameter to control the surface reflectivity in high reflectivity semiconductor materials. The use of a reverse patterning is simpler than positive patterning using a negative resist and hard mask. These types of structures can be applied for enhanced light extraction in light-emitting diodes or improved light trapping in solar cells. We have also carried out FDTD simulations to show the possible applications of the patterned interface towards light trapping in solar photovoltaics showing a maximum absorption power of 83% over a broad wavelength of 300–800 nm This technique can also be applied to other semiconductor materials to achieve highly efficient optoelectronic devices for broadband applications.
